# Prevalence and Correlates of Individuals Screening Positive for Depression and Anxiety on the PHQ-4 in the German General Population: Findings from the Nationally Representative German Socio-Economic Panel (GSOEP)

**DOI:** 10.3390/ijerph17217865

**Published:** 2020-10-27

**Authors:** André Hajek, Hans-Helmut König

**Affiliations:** Department of Health Economics and Health Services Research, University Medical Center Hamburg-Eppendorf, 20246 Hamburg, Germany; h.koenig@uke.de

**Keywords:** anxiety, depression, general population, mental disorders, Patient Health Questionnaire (PHQ), prevalence, SOEP, mental health

## Abstract

Our aim was to estimate the prevalence and correlates of probable depression and anxiety in the general adult population in Germany. Repeated cross-sectional data (i.e., cross-sectional data observed at different time points: year 2012 and year 2014) were derived from the innovation sample of the German Socio-Economic Panel, a population-based study of German households. The validated Patient Health Questionnaire (PHQ-4) was used to measure probable depression and anxiety. In the analytical sample, *n* equaled 2952 individuals. According to the PHQ-4 cut-off values, 10.4% of the individuals had probable depression and 9.8% of the individuals had probable anxiety. Regressions revealed that the likelihood of depression was positively associated with lower age (OR: 0.98 (95% CI: 0.98–0.99)), being unmarried (and living together with spouse) (OR: 0.75 (0.58–0.98)), worse self-rated health (OR: 1.99 (1.73–2.27)), and more chronic diseases (OR: 1.18 (1.07–1.31)). Furthermore, the likelihood of anxiety was positively associated with being female (OR: 1.36 (95% CI: 1.04–1.76)), lower age (OR: 0.98 (95% CI: 0.97–0.99)), low education (medium education, OR: 0.69 (0.50–0.95)), worse self-rated health (OR: 2.00 (1.74–2.30)), and more chronic diseases (OR: 1.15 (1.03–1.27)). The magnitude of depression and anxiety was highlighted. Clinicians should be aware of the factors associated with probable depression and anxiety.

## 1. Introduction

Mental disorders can be defined as a behavioral or mental pattern that contributes to the distress or impairment of personal functioning [1]. They are a significant public health issue [2,3]. More precisely, national surveys in Europe [4] and the United States [5] have demonstrated that depression and anxiety are frequent mental disorders and are associated with subsequent functional decline [6], frailty [7], or cardiovascular diseases [8]. Both are also associated with a tremendous economic burden [9,10,11], e.g., in terms of sick leave days [12]. Furthermore, both are associated with increased loneliness [13]. Moreover, they are associated with lower satisfaction with life [14], lower general self-esteem [15], and lower optimism [16]. Additionally, individuals with these mental disorders often face stigmatization [17,18].

Therefore, and rather unsurprisingly, various studies exist focusing on numerous other factors associated with depression or anxiety (e.g., [19,20,21]). Moreover, some nationally representative studies exist that present data on the prevalence of depression and anxiety in the general adult population and identify the correlates of them (for example, [22,23,24,25,26]). A few studies based on data from the general adult population in Germany also exist [27,28]. However, these studies only rely on moderate response rates (ranging from 42% to 62%) [27,28]. Therefore, the aim of this study was to estimate the prevalence rates and correlates of probable depression and anxiety based on data from a nationally representative sample in Germany with very high response rates.

Knowledge about the correlates of depression and anxiety is important to identify individuals at risk. This may be important for, among others, clinicians and mental health professionals. Going further, early detection and treatment of these disorders may help to reduce the psychosocial (e.g., in terms of lower life satisfaction, lower self-esteem, increased loneliness, and lower health-related quality of life) and economic burden (e.g., in terms of sick leave days) associated with these mental disorders.

## 2. Materials and Methods

### 2.1. Sample

Data were taken from the nationally representative German Socio-Economic Panel (GSOEP), located at the German Institute for Economic Research, DIW Berlin, which started in 1984. The GSOEP is a widely used long-running household panel study. It solely uses random probability samples (drawing from a nation-wide two-stage stratified sampling procedure) [29]. The nation-wide sample points are sampled by federal, state, and municipality size in a first step. In a second step, households are sampled using a random walk procedure within each sample point. Further details are provided elsewhere [29].

Each year, approximately 11,000 households and more than 20,000 individuals take part in the interviews. Various topics are included in the GSOEP, such as occupational history, health, and wellbeing. Very high response rates [30] and low attrition rates [31] have been demonstrated for the GSOEP. Concerning the GSOEP study, further details have been given elsewhere [32].

The Patient Health Questionnaire (PHQ-4), our outcome measure, was exclusively quantified in the innovation sample from the GSOEP (also called the GSOEP-IS) in the years 2012 and 2014. Comparable to the GSOEP, the GSOEP-IS is also a representative sample covering the community-dwelling adult population [33]. Since we focused on depression and anxiety, repeated cross-sectional data (i.e., cross-sectional data observed at different time points) were taken from the GSOEP-IS from the years 2012 and 2014 (for reasons of data availability). This means individuals in the year 2012 are different from those in the year 2014. Thus, in contrast to panel data, the same individuals are not followed over time and, consequently, individual trajectories are not available. However, using repeated cross-sectional data can assist in determining changes at the aggregate level for populations. In our analytic sample, *n* equaled 2952 individuals. The GSOEP-IS covers core questions (e.g., sociodemographic characteristics) and additionally includes innovative content which is solely designed by the users. Based on a highly competitive process, the tools are selected.

Informed consent was given by all participants in the GSOEP. Since the criteria for the need of an ethical statement were not fulfilled (e.g., examination of patients or risk for the respondents), an ethical approval was not obtained. Nevertheless, the German Council of Science and Humanities (Wissenschaftsrat) evaluated and approved the German Socio-Economic Panel (GSOEP).

### 2.2. Dependent Variables

A self-reported questionnaire, including the 2-item Patient Health Questionnaire (PHQ-2 [34,35]) and the 2-item Generalized Anxiety Disorder screening tool (GAD-2 [36]), was used. The GAD-2 covers the two main criteria for GAD, which have been demonstrated to be effective screening tools for panic disorder (sensitivity: 0.76; specificity: 0.81), post-traumatic stress disorder (sensitivity: 0.59; specificity: 0.81), and social anxiety (sensitivity: 0.70; specificity: 0.81) [36]. The PHQ-2 covers the two Diagnostic and Statistical Manual of Mental Disorders (DSM-V) diagnostic main criteria for depressive disorders [37]. Combining these two-item measures results in a composite four-item scale, the so-called Patient Health Questionnaire-4 (PHQ-4). The items are as follows: Over the last 2 weeks, how often have you been bothered by any of the following problems? (1 (Not at all), 2 (Several days), 3 (More than half the days), 4 (Nearly every day)).

- Little interest or pleasure in doing things
- Feeling down, depressed, or hopeless
- Nervousness, anxiety, or tension
- Not be able to stop or control worrying

Items were recoded from 1–4 to 0–3. For both PHQ-2 and GAD-2, sum scores of ≥3 were proposed as cut-off points for probable depression or anxiety, respectively [35,36]. Further details regarding the PHQ-4 are provided elsewhere [38,39]. In the current study, Cronbach’s alpha for PHQ-4 was 0.81, which suggested a good internal consistency of this tool.

### 2.3. Independent Variables

In regression analysis, socioeconomic independent variables were included as follows: age, sex, family status (married, and living together with spouse; married, and living separated from spouse; widowed; single; divorced), educational level (International Standard Classification of Education (ISCED-97) [40]; low education (ISCED 0–2), medium education (ISCED 3–4), and high education (ISCED 5–6)), and labor force participation (not employed; employed (including: full-time employed; regular part-time employed; vocational training; marginally employed; near retirement, zero working hours; military service; community service; sheltered workshop)).

In addition, two health-related variables were included in regression analysis: self-rated health (which ranged from 1 (very good) to 5 (very bad)) as well as the number of chronic conditions (count score including: diabetes; asthma; cardiac disease (also: cardiac insufficiency, weak heart); cancer; stroke; migraine; high blood pressure; dementia; joint diseases (including arthritis/rheumatism); chronic back pain; sleep disorder; other illness).

### 2.4. Statistical Analysis

First, sample characteristics were provided. After that, the results of prevalence estimates (for probable depression and probable anxiety) by sex, age, and education were computed (sample weights were used). Subsequently, the correlates of depression and anxiety were identified using multiple logistic regressions. It is worth noting that data from both years (year 2012 and year 2014) were pooled in our study. Please see Brüderl and Ludwig [41] for further details regarding pooled regression analysis. Different individuals filled out the PHQ-4 in the years 2012 and 2014.

In a robustness check, the conventional multiple logistic regressions were replaced using penalized maximum likelihood logistic regressions. In our study, the level of significance was set at α = 0.05. Stata 16.0 was used to conduct statistical analysis.

## 3. Results

### 3.1. Sample Characteristics

Sample characteristics for our analytical sample (*n* = 2952; (year 2012 and year 2014 were pooled)) are shown in Table 1. In our analytical sample, average age was 51.3 years (SD: 17.7 years), 51.9% of the individuals were female, and 60.5% of the individuals had a medium education. Furthermore, 56.8% of the individuals were married and living together with their spouse. Further details are given in Table 1.

### 3.2. Prevalence of Probable Depression and Anxiety Stratified by Sex, Age, and Education

According to the PHQ-4 cut-off values, 10.4% of the individuals had probable depression and 9.8% of the individuals had probable anxiety. More precisely, 85.1% of the individuals had both no probable depression and no probably anxiety. Furthermore, 5.1% of the individuals had probable depression only, 4.5% of the individuals had probable anxiety only, and 5.3% had both probable depression and probable anxiety. In further detail, prevalence estimates for probable depression and probable anxiety stratified by sex, age, and education are provided in Table 2.

### 3.3. Regression Analysis

Findings of multiple logistic regressions are presented in Table 2. Pseudo R² values were 0.10 (with depression as outcome measure) and 0.11 (with anxiety as outcome measure). In our study, variance inflation factors (VIFs) were rather low (mean VIF was 1.29; highest VIF was 1.55), which showed that multicollinearity was not a threat to the validity of our results.

Multiple logistic regressions revealed that the likelihood of depression was positively associated with lower age (OR: 0.98 (95% CI: 0.98–0.99)), not being married (and living together with spouse) (OR: 0.75 (0.58–0.98)), worse self-rated health (OR: 1.99 (1.73–2.27)), and more chronic diseases (OR: 1.18 (1.07–1.31)). Furthermore, the likelihood of anxiety was positively associated with being female (OR: 1.36 (95% CI: 1.04–1.76)), lower age (OR: 0.98 (95% CI: 0.97–0.99)), low education level (medium education, OR: 0.69 (0.50–0.95)), worse self-rated health (OR: 2.00 (1.74–2.30)), and more chronic diseases (OR: 1.15 (1.03–1.27)) (Table 3).

In our sensitivity analysis, conventional logistic regressions were replaced by penalized maximum likelihood logistic regressions (results not shown, but available upon request). However, in terms of significance and effect sizes, results remained virtually the same.

## 4. Discussion

### 4.1. Main Findings

Based on nationally representative data, the purpose of our study was to present data on the prevalence of depression and anxiety and to determine the correlates of them. We showed that both depression and anxiety are common in the adult population in Germany (10.4% of the individuals had probable depression and 9.8% of the individuals had probable anxiety). Regression showed that the likelihood of depression was positively associated with lower age, not being married, worse self-rated health, and more chronic diseases. Similarly, the likelihood of anxiety was positively associated with being female, lower age, low educational level, worse self-rated health, and more chronic diseases.

### 4.2. Previous Research and Possible Explanations

Using data from a nationally representative sample, this study adds knowledge on the prevalence of depression and anxiety in Germany. Additionally, factors associated with them were identified. We showed that probable depression and anxiety are frequent in the adult population. With regard to Germany, similar prevalence rates have been reported. More precisely, for the adult population (18 to 79 years old) in Germany, slightly lower prevalence rates for depression (PHQ-9 ≥ 10 points) have been reported (8.1%) in a previous study [27], based on data from the German Health Interview and Examination Survey for Adults (DEGS). In another study also based on DEGS data (adult (18 to 79 years old) population in Germany) and using a diagnostic interview, a slightly higher prevalence rate has been documented for anxiety disorders (15.3%; during the past 12 months; based on the Composite International Diagnostic Interview (CIDI)/DSM-IV-TR) [28]. This survey was carried out from 2009 to 2012. International studies based on nationally representative samples of the adult population mainly reported similar prevalence rates for anxiety disorders (for example: United States, individuals aged 18+ years: 11.1%; Iran, individuals aged 15 to 64 years: 15.6% [24]), and depression (for example: Greece, individuals aged 18 to 79 years: 12.3%; [22]; Hong Kong, individuals aged 18+ years: 7.0% [26]). It should be acknowledged that somewhat higher (for example, in France, individuals aged 18+ years, anxiety disorder: 21.6% [25]) or lower prevalence rates (for example, in Israel, individuals aged 21+ years, anxiety disorder: 3.2% [42]) have also been documented in some studies based on nationally representative samples. Generally, we think that the differences in the prevalence rates reported in our study and the existing German and international studies can be explained by the use of different tools [43], diagnostic thresholds [44], and—in the case of international studies—by differences in the cultural background [45]. A previous systematic review and meta-analysis focusing on the global prevalence of anxiety disorders also showed that methodological differences and cultural factors account for a great proportion of variability in prevalence rates [46]. The importance of cultural aspects in social anxiety disorders has also been highlighted by Hofmann et al. [47].

In our study, factors associated with probable depression and anxiety such as being female, lower age, being unmarried, and having worse health are mainly in line with previous studies [43]. For example, it has been demonstrated in other studies that depression and anxiety are both associated with low self-rated health and morbidity [48,49]. With regard to underlying mechanisms, previous studies have shown that, among other things, factors such as insomnia or stress can mediate the link between depression and cardiovascular diseases [50].

In line with our study, a link between marital status and depression has been shown in previous research [51]. This supports the protection/support hypothesis. According to this hypothesis, individuals who are married report better (physical and) mental health compared to unmarried individuals, because they have the continuous companionship of a spouse. A spouse provides emotional gratification, interpersonal closeness, and assistance in dealing with stress [51].

Given the fact that previous research showed a strong link between employment status and depression [52], at first glance, it was surprising that both depression and anxiety were not associated with employment status in our study. However, this may be explained by the fact that our variable to quantify employment status included a large variety of employed individuals (ranging from full-time employed, over regular part-time employed to marginally employed). Future research based on nationally representative samples is required to clarify the link between employment status and depression as well as anxiety.

Other studies have also shown that the prevalence of depression rises in young adults, is highest in middle-aged individuals (of 50 to 69 years), and gradually declines in older individuals [27,53]. When we added quadratic and cubic age terms to additional logistic regression models (not shown here, but available upon request) in the current study, linear, quadratic, and cubic age terms were statistically significant (both with depression and anxiety as outcome measures). Within this context, it is worth noting that individuals aged 80 years and over had markedly higher prevalence rates for both probable depression (21.8%) and anxiety (16.9%) in our study. Nevertheless, with regard to anxiety, quite similar prevalence rates (14.5%) were reported for individuals aged 82 years and over in Germany based on the multicenter prospective cohort study AgeCoDe/AgeQualiDe [54]. Another study based on the AgeCoDe cohort showed prevalence rates (depression if 15-item version of the Geriatric Depression Scale ≥ 6 [55]) of 10.1% among individuals aged 80 to 84 years and 13.7% among individuals aged 85 years and over [56].

Quite unexpectedly, being female was not associated with the likelihood of depression. This may reflect the fact that the attitude toward mental disorders may have changed over the past decades—particularly in men. While especially men may have underreported mental disorders in the past (e.g., due to stigmatization), they may have more truly self-evaluated their current mental health status in questionnaires in the past few years [57]. However, further research is required to gain more insights into this link.

### 4.3. Strengths and Limitations

Our current study extends the knowledge about the prevalence of probable depression and anxiety and their correlates in Germany. In our study, data were taken from a population-based sample, the GSOEP-IS. Furthermore, in contrast to former German studies [27,28], very high response rates have been documented for the GSOEP [30]. For example, annual response rates were commonly over 90% [58]. The PHQ-4 is a four-item screening scale for depression and anxiety shown to have good psychometric characteristics [34,35,36,38,39]. However, it has recently been shown that, for example, the GAD-7 is superior compared to the GAD-2 with regard to several psychometric characteristics [59]. Moreover, this is a repeated cross-sectional study, which has its acknowledged limitations, such as the inability to observe individuals over time. Therefore, future longitudinal studies are required to overcome these shortcomings. Furthermore, further studies are required to determine the prevalence rates of depression and anxiety in institutionalized settings in Germany. Another limitation of this study is that there was a lack of clinical variables. More precisely, future studies are required that include variables such as selective serotonin reuptake inhibitors (SSRIs) or benzodiazepines.

## 5. Conclusions

Clinicians should be aware of the factors associated with probable depression and anxiety. This may also help with early detection of depression and anxiety. Longitudinal studies are required to gain further insights.

## Figures and Tables

**Table 1:** Sample characteristics for analytical sample (GSOEP-IS, 2012 and 2014; *n* = 2952).

Variables	Mean (SD)/N (%)
Age: Mean (SD)	51.3 (17.7)
Gender: N (%)	
Male	1421 (48.1%)
Female	1531 (51.9%)
Education (according to the ISCED-97 classification): N (%)	
Low education (ISCED-97: 0–2)	455 (15.4%)
Medium education (ISCED-97: 3–4)	1785 (60.5%)
High education (ISCED-97: 5–6)	712 (24.1%)
Marital status: N (%)	
Married, living together with spouse	1678 (56.8%)
Other (married, not living together with spouse; divorced; widowed; single)	1274 (43.2%)
Employment status: N (%)	
Employed (full-time employed; regular part-time employed; vocational training; marginally employed; near retirement, zero working hours; military service; community service; sheltered workshop)	1639 (55.5%)
Not employed	1313 (44.5%)
Self-rated health (from 1 = “very good” to 5 = “very bad”): Mean (SD)	2.5 (1.0)
Sum of chronic diseases: Mean (SD)	1.0 (1.2)

**Table 2:** Prevalence estimates for probable depression and probable anxiety stratified by sex, age, and education (weighted proportions and 95% confidence intervals in parentheses).

	Absence of Probable Depression	Presence of Probable Depression	Absence of Probable Anxiety	Presence of Probable Anxiety
Total	89.6 (88.3–90.8)	10.4 (9.2–11.7)	90.2 (88.9–91.3)	9.8 (8.7–11.1)
Gender				
Male	90.5 (88.8–92.1)	9.5 (7.9–11.3)	92.6 (91.0–94.0)	7.4 (6.0–9.0)
Female	88.8 (86.8–90.5)	11.2 (9.5–13.2)	88.0 (86.0–89.7)	12.0 (10.3–14.0)
Age group				
17 to 29 years	89.7 (85.9–92.6)	10.3 (7.4–14.1)	88.6 (84.8–91.6)	11.4 (8.4–15.2)
30 to 39 years	89.1 (85.0–92.2)	10.9 (7.8–15.0)	90.0 (86.2–92.9)	10.0 (7.1–13.8)
40 to 49 years	91.6 (88.5–93.9)	8.4 (6.1–11.5)	89.8 (86.5–92.4)	10.2 (7.6–13.5)
50 to 59 years	87.9 (84.7–90.6)	12.1 (9.4–15.3)	89.3 (86.2–91.8)	10.7 (8.2–13.8)
60 to 69 years	90.8 (87.4–93.3)	9.3 (6.7–12.7)	91.8 (88.9–94.0)	8.2 (6.0–11.1)
70 to 79 years	91.9 (88.4–94.4)	8.1 (5.6–11.6)	94.6 (91.6–96.5)	5.4 (3.5–8.4)
80 years and over	78.2 (68.9–85.3)	21.8 (14.7–31.1)	83.1 (75.0–88.9)	16.9 (11.1–25.0)
Education (ISCED-97)				
Low education (ISCED-97: 0–2)	84.9 (80.5–88.5)	15.1 (11.5–19.5)	84.0 (79.8–87.6)	16.0 (12.4–20.2)
Medium education (ISCED-97: 3–4)	89.9 (88.2–91.3)	10.1 (8.7–11.8)	90.8 (89.2–92.1)	9.2 (7.9–10.8)
High education (ISCED-97: 5–6)	92.2 (89.7–94.1)	7.8 (5.9–10.3)	92.9 (90.5–94.7)	7.1 (5.3–9.5)

**Table 3:** Determinants of probable depression and probable anxiety.

Independent Variable	Depression	Anxiety
Gender: Female (Ref.: Male)	0.93 (0.72–1.19)	1.36 * (1.04–1.76)
Age	0.98 *** (0.98–0.99)	0.98 *** (0.97–0.99)
Employment status: Not employed (Ref.: Employed)	1.30+ (0.97–1.74)	1.27 (0.94–1.70)
Educational level (ISCED-97 classification): Medium education (Ref.: low education)	0.85 (0.61–1.18)	0.69 * (0.50–0.95)
High education	0.82 (0.54–1.24)	0.67+ (0.44–1.02)
Marital status: Married, living together with spouse (Ref.: Other)	0.75 * (0.58–0.98)	0.79+ (0.61–1.03)
Self-rated health (from 1 = “very good” to 5 =“very bad”)	1.99 *** (1.73–2.27)	2.00 *** (1.74–2.30)
Count score of chronic diseases	1.18 ** (1.07–1.31)	1.15 * (1.03–1.27)
Constant	0.04 *** (0.02–0.07)	0.04 *** (0.02–0.08)
Observations	2952	2957
Pseudo R^2^	0.103	0.108

Comments: Odds Ratios were reported; 95% confidence intervals in parentheses; *** *p* < 0.001, ** *p* < 0.01, * *p* < 0.05, + *p* < 0.10; 0 = absence of probable depression, 1 = presence of probable depression; 0 = absence of probable anxiety, 1 = presence of probable anxiety. The marital status “Other” included (i) married, and living separated from spouse, (ii) widowed, (iii) single, and (iv) divorced. The occupational status “Employed” included (i) full-time employed, (ii) regular part-time employed, (iii) vocational training, (iv) marginally employed, (v) near retirement, (vi) zero working hours, (vii) military service, (viii) community service, and (ix) sheltered workshop.
